# The application of metaverse in occupational health

**DOI:** 10.3389/fpubh.2024.1396878

**Published:** 2024-04-11

**Authors:** Yufu Tang, Hongying Liang, Jingming Zhan

**Affiliations:** Division of Radiology and Environmental Medicine, China Institute for Radiation Protection, Taiyuan, China

**Keywords:** metaverse, occupational health, virtual reality, artificial intelligence, risk assessment, telemedicine

## Abstract

**Background:**

The metaverse, as a new digital interactive platform, is garnering significant attention and exploration across industries due to technological advancements and societal digital transformation. In occupational health, there is immense potential for leveraging the metaverse to enhance work environments and occupational health management. It offers companies more efficient and intelligent solutions for occupational health management while providing employees with safer and more comfortable work environments.

**Methods:**

A comprehensive literature search was conducted using PubMed, Web of Science, IEEE Xplore, and Google Scholar databases to identify relevant studies published between January 2015 and March 2024. The search terms included “metaverse,” “virtual reality,” “occupational health,” “workplace safety,” “job training,” and “telemedicine.” The selected articles were analyzed, and key findings were summarized narratively.

**Results:**

The review summarizes the broad application prospects of metaverse technology in immersive training, occupational risk identification and assessment, and occupational disease monitoring and diagnosis. However, applying the metaverse in occupational health also faces challenges such as inadequate technical standards, data privacy issues, human health hazards, high costs, personnel training, and lagging regulations.

**Conclusion:**

Metaverse offers new possibilities for addressing the numerous challenges faced in occupational health and has broad application prospects. In the future, collaborative efforts from multiple stakeholders will be necessary to promote the sustainable development of metaverse technology in occupational health and better protect workers’ occupational health.

## 1 Introduction

The metaverse, a digital shared space that integrates cutting-edge technologies such as virtual reality (VR), augmented reality (AR), artificial intelligence (AI), and blockchain, has the potential to revolutionize various industries. The term “metaverse” was first coined by Neal Stephenson in his 1992 science fiction novel “Snow Crash,” where it was described as a virtual world that exists parallel to the real world, constantly online and influenced by real-world events (1). As computer technology rapidly advances, the metaverse gradually transitions from a science fiction concept to reality, bringing new opportunities to various sectors. In education, immersive learning environments based on VR and AR technologies can provide learners with an engaging and interactive experience, enhancing learning outcomes and participation (2). In clinical practice, medical professionals can utilize virtual simulation technology for surgical rehearsals and skill training, reducing risks and shortening learning curves (3). These application cases demonstrate the immense potential of metaverse technology to revolutionize traditional industries. Occupational health, a critical field concerning the health and safety of the working population (4), is also in urgent need of exploring the applications of the metaverse.

Traditional occupational health practices have room for improvement, such as occupational training, occupational risk identification and assessment, and occupational disease detection and diagnosis. Occupational health training is monotonous, disconnected from reality, lacks immersive experiences, and fails to meet individualized needs. Occupational risk identification and assessment rely on manual surveys, which are inefficient and lack real-time dynamic monitoring and early warning capabilities, resulting in non-intuitive assessment results. Identifying and diagnosing occupational diseases need more objective quantitative indicators, and early symptoms are easily overlooked. Moreover, diagnostic and treatment resources are unevenly distributed, and the medical process is cumbersome (5–7).

Introducing metaverse technology into occupational health may help solve the above problems and open up new paths for occupational health. First, developing immersive and interactive occupational health education courses using AR/VR technologies can provide personalized guidance and significantly increase employees’ learning interest and proactivity (8). Second, utilizing VR to construct realistic occupational scenarios can simulate various occupational hazards (9), providing accurate and intuitive data support for occupational health monitoring and risk assessment. Finally, based on digital twin (DT), real-time monitoring and management of the workplace and employees’ health status can be realized (10), significantly improving the effectiveness of occupational health management. Through remote medical platforms, occupational disease patients can access diagnostic and treatment services more conveniently, increasing the effectiveness of occupational disease prevention and control.

In summary, metaverse technology has broad application prospects in occupational health. It has the potential to break through the limitations of traditional occupational health models and open up new paths for occupational health. The application of the metaverse in occupational health is still in its infancy, facing many challenges in crucial technology breakthroughs, industry solution development, establishment of standards and norms, and improvement of safety assurance mechanisms. In the future, it will be necessary to strengthen the adaptation and optimization of metaverse technologies in occupational health scenarios, develop verticalized industry solutions targeting the specific needs of occupational health management and services to improve the pertinence and effectiveness of metaverse applications and establish and improve the standards, norms, and safety assurance mechanisms for metaverse applications to foster a great industry application ecosystem. Through continuous research and practice in the above areas, the metaverse will undoubtedly propel the field of occupational health to reach new heights, promoting the intelligent, refined, and personalized development of occupational health and opening a new chapter in occupational health. This review will comprehensively analyze the current applications and challenges of crucial metaverse technologies in occupational health, aiming to provide references and insights for relevant research and practice.

## 2 The technological characteristics of the metaverse

“Metaverse” is a combination of the prefix “Meta” (meaning beyond) and “Verse” (meaning universe), implying that the metaverse is a new world expressed through the internet and other digital media. There is currently no standard definition of the metaverse, but a widely recognized definition by Prof. Dr. Markus Weinberger from Aalen University of Applied Science (11) is: “The metaverse is an interconnected web of ubiquitous virtual worlds partly overlapping with and enhancing the physical world. These virtual worlds enable users who are represented by avatars to connect and interact with each other and to experience and consume user-generated content in an immersive, scalable, synchronous, and persistent environment.”

The metaverse has three main characteristics: (1) multi-technology convergence; (2) sociality; (3) hyper-spatial-temporality. As a new Internet application, the metaverse integrates a variety of new technologies and has the characteristic of multi-technology convergence; as a new social form, the metaverse has the characteristic of sociality; as a virtual world that is parallel and closely related to the real world, the metaverse has the characteristic of hyper-spatial-temporality (12). The metaverse is the inevitable trend of social informatization and virtualization and can be considered the ultimate stage of Internet development.

“4R” includes VR, AR, mixed reality (MR), and extended reality (XR), collectively representing a new era of digital and real-world interactions. In this new era, VR allows users to immerse themselves in environments completely constructed by digital technology, and experience a sensation akin to being in real-life situations; AR overlays two-dimensional or three-dimensional virtual images onto the real world, providing an enhanced perceptual experience; MR goes further, merging elements of the real world with information from the virtual world, creating a new hybrid reality space where reality and virtuality can interact and integrate (13). XR is an umbrella term for these concepts, including VR, AR, MR, and other potential new forms of reality, offering a broader platform for virtual interactions. In industries like healthcare, the application of XR technology is seen as a key to innovating social and industrial ecosystems. Through simulating real medical situations, offering AR-based complex surgery visual aids, and using MR for medical education and patient care, XR technology is reshaping the future of the medical industry (14–16). The combined application of these technologies not only enhances the quality and accessibility of medical services but also improves the interaction efficiency between patients and medical professionals. With continuing technological advancements, the metaverse is leading a profound transformation in occupational health.

## 3 Applications of the metaverse in occupational health

### 3.1 Immersive training

Traditional training and education face geography, economic conditions, teacher quality, and time limitations. The effective integration of the metaverse with occupational training allows for the simulation of the workplace. It enables the incorporation of real-world elements such as educational content, teaching methods, and role relationships. This transcends the constraints of physical space and conditions, enabling cross-temporal and cross-spatial experiential learning. For example, doctors can use advanced 3D imaging and motion capture technology to demonstrate standard surgical procedures and teach textbook concepts through virtual scenarios. Students can perform thorough dissections without risk (3). The metaverse combines theory and practice, addressing the practical shortcomings of remote education. Lee et al. (17) integrated the metaverse into an aircraft maintenance course, safely simulating high-cost and high-risk scenarios while allowing for repeated practice of complex and challenging tasks. Using 3D modeling tools, the metaverse can construct highly realistic underground coal mine models connected to various underground sensors to understand real-time conditions. This accurately reflects the actual underground situation in the metaverse scene, and disaster simulation scripts can be established using big data algorithms. Participants can experience these imagined disaster scenarios directly, enhancing their psychological resilience and adaptability when facing emergencies (18). Through technologies such as VR, AR, and sensors, the metaverse can create precise replicas of multi-story building structures and simulate fire scenarios (19). In this virtual world, participants wearing VR headsets can experience authentic fire conditions such as smoke spread, obstructed vision, and auditory confusion. Firefighters can improve fire response abilities through virtual training. At the same time, architects and engineers can utilize simulation data to refine building designs and ensure safety.

From the above analysis, it is evident that by constructing realistic virtual work environments, simulating various occupational hazards, and providing interactive operation training and multi-person collaborative drills, the metaverse compensates for the practical limitations of traditional occupational training. It creates a safe, authentic, and engaging learning environment for trainees. The learning approach significantly enhances trainees’ learning interest and participation, deepening their understanding and mastery of knowledge and skills, thus achieving better training outcomes. However, developing high-quality occupational health training content requires substantial investment in human and material resources, placing higher demands on instructional design and development teams.

### 3.2 Occupational risk identification and assessment

Risk assessment in occupational health is a critical task aimed at identifying, analyzing, and controlling potential hazards in the workplace. Traditional methods rely on on-site inspections, historical data analysis, and expert experience. However, physical conditions, time, and economic costs often limit these methods. The metaverse can simulate various work scenarios and environments through VR and AI, identify hazardous factors, quantitatively analyze risks using AI algorithms, and improve occupational health management. The following three examples illustrate how the metaverse identifies and assesses occupational risks and investigates the feasibility and challenges of its implementation.

1. Emergency Response: For emerging hazards, the metaverse enables efficient rescue operations while ensuring the safety of rescue personnel (20). For instance, when a chemical plant explosion occurs, identifying hazardous chemical substances, determining threat types, and monitoring dosage are fundamental to occupational emergency response. Facing an unknown accident scene, if rescue personnel hastily enter the chemical plant to carry out operations, they are highly likely to suffer severe physical harm or even endanger their lives. The metaverse utilizes AR to visualize the distribution of chemical substance doses, displaying gases invisible to the naked eye in different colors according to their hazard levels (21). It assesses on-site dangers and plans rescue routes for rescue personnel. Simultaneously, it identifies the types of chemical substances, assisting in selecting appropriate shielding materials and detectors and providing comprehensive protection for rescue operations.
2. Exposure to Radioactive Drugs: Nuclear medicine personnel frequently come into contact with radioactive drugs, facing the risk of radiation exposure. The metaverse allows virtual avatars to enter wards and perform remote drug administration with the assistance of AI, avoiding damage caused by radioactive substances. Moreover, through VR simulation, AR visualization, and AI analysis, it identifies potentially inhaled, ingested, or skin-contacted radioactive materials, thereby reducing the risk of internal exposure (22).
3. Mine Working Environment Monitoring: The metaverse, combined with the Internet of Things (IoT) intelligent helmets, can monitor the working environment in real-time (23), making decisions based on changes in environmental factors, significantly reducing the risk of accidents in the workplace. On this basis, through a virtual sensor network, the metaverse utilizes 3D modeling tools to construct high-fidelity underground coal mine models, create virtual scenes, and connect various underground sensors to understand the actual underground conditions in real time. This information is accurately reflected in the metaverse scene, realizing real-time monitoring and visualization of occupational hazards (24).

The above cases demonstrate that metaverse technology has vast application prospects in occupational risk identification and assessment, significantly enhancing the precision and effectiveness of risk control. The industrial sector has a solid foundation for digital transformation, and metaverse technology is becoming increasingly mature, providing necessary data and technical support. However, it also faces challenges such as complex and changing industrial scenarios, standardization, and personnel capabilities. In the future, cross-disciplinary collaborative efforts should be strengthened, and a sound occupational safety management system should be established in the metaverse environment to promote the deep integration of new technologies and traditional industries, providing solid support for occupational health.

### 3.3 Occupational disease monitoring and diagnosis

From 2009 to 2018, China officially confirmed more than 270,000 cases of occupational diseases (25), indicating a rather severe overall situation of occupational diseases. Ensuring the health of the occupational population and effectively monitoring occupational diseases are urgent tasks. Traditional methods for occupational disease monitoring and diagnosis, such as questionnaire surveys, on-site inspections, and occupational health examinations (26), make it difficult to comprehensively and dynamically grasp occupational hazards’ exposure levels and health impacts. As an immersive digital shared space that integrates emerging technologies such as VR, AR, and DT, metaverse technology provides a new approach to address these challenges.

Metaverse technology has broad application prospects in occupational disease monitoring and diagnosis, mainly reflected in the following aspects:

VR technology can simulate the impact of occupational hazards on the human body, assessing health risks at different exposure doses, frequencies, and durations (27). Additionally, through the remote medical platform built by the metaverse, patients can engage in face-to-face communication and treatment with doctors in virtual clinics from their homes or workplaces, saving time and energy spent on commuting and improving the convenience of medical consultations (28).

AR technology can assist doctors in comprehensively and accurately understanding the condition of occupational disease patients. Researchers use AR technology to merge patients’ imaging data (such as X-rays and CT scans) with anatomical structures, generating intuitive and three-dimensional visualization models (29). Doctors can observe the specific location, size, and morphology of lesions inside patients’ bodies through AR devices (such as AR glasses) and perform real-time measurements and annotations. Simultaneously, AR technology can integrate information such as patients’ occupational history and exposure history, dynamically presenting the correlation between occupational hazards and disease occurrence and development (30). Zhang et al. (31) developed an intelligent system for pneumoconiosis diagnosis that integrates patients’ chest CT images and occupational exposure information, generating intuitive three-dimensional visualization models and providing quantitative diagnostic indicators, and the system outperformed radiologists in the accuracy of pneumoconiosis staging.

DT technology can achieve full-cycle and full-process management from occupational hazards to the health status of the occupational population. Researchers collect physiological parameters, behavioral data, occupational exposure data, and other information from the occupational population to construct personalized digital human models (32). Furthermore, DT can integrate multi-source heterogeneous data, such as occupational health examination data, to achieve early warning and intelligent diagnosis of occupational diseases. For example, Jia et al. (33) utilized DT to construct a comprehensive, intelligent prevention, diagnosis, and treatment solution for occupational diseases in the petrochemical industry based on extensive data analysis and continuous improvement of occupational health management.

The application of metaverse blockchain in occupational health has opened up new horizons for data management and enhanced the efficiency and transparency of public health supervision. This technology allows individuals’ identities and health monitoring data to be securely stored and tracked (34). When an individual’s health status needs to be recorded, the relevant information is directly registered in the corresponding blockchain block, achieving real-time and accurate information access. This allows for swift access to an individual’s latest health data and enables comparison with their historical health records.

In summary, metaverse technology demonstrates innovative potential in occupational disease monitoring and diagnosis. VR technology simulates occupational hazards, enabling real-time monitoring and health risk assessment. AR technology assists doctors in deeply understanding patients’ conditions, improving diagnostic accuracy and efficiency. DT technology predicts occupational disease risks through digital models and integrates data for early warning and intelligent diagnosis. When applied to data management, blockchain technology enhances the efficiency of public health supervision. These technologies provide novel means for occupational disease monitoring, diagnosis, and prevention.

## 4 Challenges and countermeasures of the metaverse in occupational health

Despite the promising prospects of the metaverse in the field of occupational health, it is essential to be aware of potential risks, which include the following aspects:

1. Inadequate Technical Standards and Specifications: There currently needs to be more unified technical standards and specifications. The technological barriers between different manufacturers and platforms lead to difficulties in interoperability and data exchange between systems (35). In occupational health, there is an urgent need to formulate industry standards for metaverse-related technologies to regulate critical aspects such as virtual scenario construction, digital human modeling, and health data collection. This ensures data quality and consistency while enhancing system openness and integration.
2. Data Security and Privacy Protection: The application of the metaverse relies on collecting and analyzing vast amounts of data involving sensitive information such as personal details, health status, and behavioral trajectories of the occupational population. Data breaches or illegal use will pose a severe threat to individual privacy and information security (36). Although blockchain technology can provide a certain level of data security, strict security protection measures and privacy protection mechanisms still need to be established at every data collection stage, transmission, storage, and access.
3. Health Risks: Immersive metaverse experiences may negatively impact users’ physical and mental health. Prolonged use of VR/AR devices can cause discomfort symptoms such as dizziness, headaches, and visual fatigue (37). It may even lead to addiction to the virtual world and alienation from reality. Moreover, harmful content such as violence and horror in virtual scenarios may adversely affect users’ mental health (38). When applying metaverse technology in occupational health, it is necessary to thoroughly assess its potential health risks, formulate reasonable usage guidelines and time limits, and strengthen the management of harmful content.
4. High Implementation Costs: The metaverse application requires substantial investments in hardware and software infrastructure, such as high-performance computing devices, high-speed network transmission, large-capacity storage, and interactive devices. Additionally, it is necessary to develop compatible software systems and applications, which also demand highly skilled technical personnel, resulting in high implementation costs (39). For many small and medium-sized enterprises, the threshold for fully implementing metaverse technology is high, especially the heavy economic burden. Therefore, reducing the implementation cost of metaverse technology and improving its economic feasibility are critical factors in promoting its widespread application in occupational health.
5. Personnel Training and Management Model Innovation: The application of metaverse technology places new demands on practitioners’ knowledge structure and skills, requiring the support of interdisciplinary personnel. On the one hand, occupational health management personnel must master cutting-edge technological knowledge such as VR and AI. On the other hand, technical personnel also need to understand occupational health-related work processes and management norms (40). The cultivation and introduction of high-quality, interdisciplinary personnel are the foundation for the practical application of metaverse technology. At the same time, applying metaverse technology also challenges traditional occupational health management models, requiring innovation and transformation in organizational structure, management processes, performance appraisal, and other aspects to establish a new management system adapted to the metaverse era.
6. Lagging Legal Regulations: Existing laws and regulations related to occupational health mainly focus on traditional occupational hazard protection and occupational disease diagnosis, with a need for more provisions for applying new metaverse technologies. There are legal gaps in data ownership, privacy protection, and liability determination. It is necessary to accelerate the revision and improvement of laws and regulations in occupational health, clarify the legal boundaries and responsibilities of metaverse technology applications, and provide legal guarantees for the application of new technologies. Additionally, law enforcement and supervision need to be strengthened to fight against illegal and non-compliant activities using metaverse technology and maintain the orderly operation of occupational health management.

In summary, applying metaverse technology in occupational health faces challenges in technology, security, cost, personnel, law, and other aspects. This requires the joint efforts of multiple parties, including the government, enterprises, research institutions, and social organizations. While solidifying the technical foundation, it is crucial to accelerate the construction of standards, specifications, laws, and regulations, optimize implementation paths and management models, and promote the deep integration of metaverse technology and occupational health management. Fully unleashing the potential and effectiveness of new technology applications will support the high-quality development of occupational health in the new era.

## 5 Conclusion

The review conducts a literature review on the current application of the metaverse in occupational health. It summarizes the broad application prospects of metaverse technology in immersive training, occupational risk identification and assessment, occupational disease monitoring and diagnosis, and other aspects, and provides new ideas for traditional occupational health management.

However, applying the metaverse in occupational health also faces challenges such as inadequate technical standards, data privacy issues, human health hazards, high costs, personnel training, and lagging regulations. In the future, the metaverse in occupational health necessitates international collaboration, standardization, practical and cost-effective solutions, industry-academia-research partnerships, interdisciplinary cooperation, and increased awareness among practitioners to ensure its safe and reliable application.

In conclusion, metaverse technology has injected new vitality and momentum into occupational health, bringing new opportunities and challenges. We call on all parties in occupational health to take active action, seize opportunities, face challenges, and jointly create a bright future for occupational health in the metaverse era.

## Author contributions

YT: Writing – original draft. HL: Writing – review & editing. JZ: Writing – review & editing.
